# The Use of 3D-Echo in Edge-to-Edge Percutaneous Tricuspid Valve Repair

**DOI:** 10.3390/jcm14030684

**Published:** 2025-01-22

**Authors:** Giulia Passaniti, Lucy M. Safi, Yoav Niv Granot, Filippo M. Sarullo, Tulio Caldonazo, Lisa Q. Rong, Corrado Fiore, Antonino Di Franco

**Affiliations:** 1 Division of Cardiology, Mount Sinai Heart Fuster Hospital, Icahn School of Medicine at Mount Sinai, New York, NY 10029, USA; giuliapassaniti94@gmail.com (G.P.);; 2 Centro Alte Specialità e Trapianti, Policlinico G. Rodolico-San Marco, University of Catania, 95123 Catania, Italy; 3 U.O.S.D. di Riabilitazione Cardiovascolare Ospedale Buccheri La Ferla Fatebenefratelli, 90123 Palermo, Italy; 4 Department of Cardiothoracic Surgery, Friedrich-Schiller-University Jena, 07743 Jena, Germany; tulio.caldonazo@med.uni-jena.de; 5 Department of Cardiothoracic Surgery, Weill Cornell Medicine, 525 E 68th St, New York, NY 10065, USA; 6 Department of Anesthesiology, Weill Cornell Medicine, New York, NY 10065, USA; 7 Department of Cardiology, Città di Lecce Hospital-GVM, 73100 Lecce, Italy

**Keywords:** tricuspid valve, echocardiography, 3D echo, TEER

## Abstract

The tricuspid valve (TV) is a complex anatomical entity. As surgical treatment for isolated tricuspid regurgitation has traditionally been associated with high peri- and post-operative mortality, advances in percutaneous transcatheter techniques of repair and replacement of the TV are emerging as safe and effective alternatives. This review summarizes the current evidence on the use of three-dimensional echocardiography to assist transcatheter-edge-to-edge repair (TEER) in patients with tricuspid regurgitation.

## 1. Introduction

First drawn in 1512 by Leonardo da Vinci [1], the tricuspid valve (TV) is a complex anatomical entity, consisting of multiple leaflets, chordae tendineae, papillary muscles, and a fibrous annulus, located between the right atrium (RA) and the right ventricle (RV). Despite its name, the TV can display numerous morphologies with a variable number of leaflets. According to the recent classification by Hahn et al., type I refers to the classical three-leaflet conformation (anterior, posterior, and septal leaflet); type II presents with a two-leaflet configuration; type III encompasses many subcategories of four-leaflet morphology, while type IV includes a TV with more than four leaflets (Table 1) [2].

The term tricuspid regurgitation (TR) refers to the inability of the TV leaflets to close properly during systole, leading to backward blood flow into the right atrium and a decrease in the forward RV stroke volume [3]. Significant TR affects more than 1.6 million people in the United States and more than 3 million in Europe, and it is associated with increased morbidity and mortality, leading to a growing interest in its management in recent years [4,5].

As surgical treatment for isolated TR has traditionally been associated with high peri- and post-operative mortality (8%) [6], advances in percutaneous transcatheter techniques of repair and replacement of the TV are emerging as safe and effective alternatives. Understanding the exact anatomy of the TV is of fundamental importance as it can affect the outcomes of the percutaneous repair procedure (e.g., a four-leaflet configuration is associated with a higher risk of residual TR, impacting patient prognosis) [7].

This review summarizes the current evidence on the use of three-dimensional echocardiography (3D-echo) to assist transcatheter-edge-to-edge repair (TEER) in patients with TR.

## 2. Etiology of TR

### 2.1. Primary TR

Primary or organic TR affects 8-10% of patients with TR, and it is due to intrinsic anatomical valve abnormalities [8]. This broad category includes congenital malformations and acquired disorders. Within congenital malformations, Ebstein’s anomaly is one of the most common causes of TR, characterized by an apical displacement and tethering of the septal and posterior leaflets [9]. Other less common congenital causes include atrioventricular canal defect, tricuspid atresia, and congenital tricuspid stenosis [10]. Acquired disorders include right-sided endocarditis (less frequent than left-sided endocarditis, but common in patients with known intravenous drug use), myxomatous degeneration, rheumatic disease, carcinoid, drug-induced disorders (e.g., fenfluramine, phentermine, methysergide, pergolide), traumas (chest wall contusion or deceleration injury trauma), and iatrogenic causes [10,11].

### 2.2. Secondary TR

**Figure 1:**
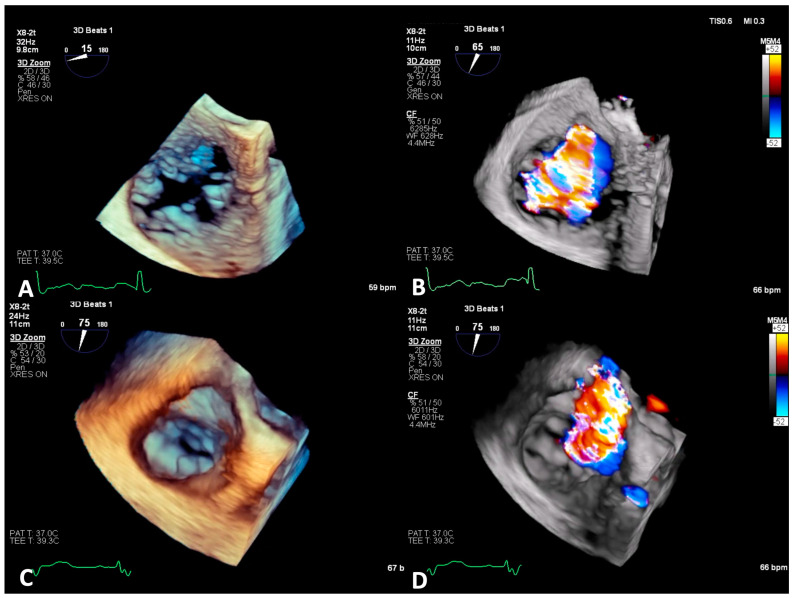
3D zoom without and with color of torrential atrial and ventricular TR. Panels (**A**,**B**): 3D zoom TEE of the TV from the mid-esophageal window, in a patient with torrential atrial TR, showing tricuspid annulus dilatation, causing a large coaptation gap, associated with a broad TR jet. Panels (**C**,**D**): 3D zoom TEE of the TV from the mid-esophageal window in a patient with ventricular TR due to tethered septal leaflet, resulting in severe TR. TEE, transesophageal echocardiography; TV, tricuspid valve; TR, tricuspid regurgitation.

Secondary or functional TR is the most common type of TR, accounting for almost 90% of severe TR causes [4]. According to the latest literature, two different phenotypes of secondary TR can be distinguished: atrial and ventricular secondary TR, each associated with different pathophysiological and prognostic characteristics (Figure 1) [12].

Atrial TR has a prevalence of 10–15% among the causes of secondary TR, and it is caused by RA enlargement, annular dilatation, and a consequent lack of leaflet coaptation in the setting of normal RV dimensions and function [13]. Longstanding atrial fibrillation is emerging as the most relevant cause of atrial TR; other risk factors include advanced age and female sex [14]. The atrial TR phenotype is characterized by RA enlargement, predominant annulus dilatation (especially posteriorly) with planar coaptation of the tricuspid leaflets, and usually preserved ventricular systolic function [15]. As RA dilatation progresses, the leaflet coaptation surface decreases with a consequent increase in the degree of TR, characterized by a mainly central jet [16].

Ventricular functional TR can be caused by left heart diseases, pulmonary hypertension, and RV dilation [17]. Left heart diseases can be due to decreased ejection fraction and valvular abnormalities (mitral and/or aortic): the left ventricle (LV) can become hypertrophic or dilated, imposing increased pressure on the pulmonary circulation and the RV. With time, increased RV afterload and preload, respectively due to increased pressure and volume, can cause RV dilatation and decreased RV function [18].

Ventricular functional TR is characterized by at least moderate RV dilatation and dysfunction, which tethers the leaflets apically, shifting the coaptation point more ventricular and causing TR [16]. In contrast to atrial TR, the remodeling pattern of the RV is spherical or elliptical due to an increase in its mid-diameter, causing apical displacement of the papillary muscles, leaflet tethering, and a central or eccentric TR jet.

### 2.3. Cardiac Implantable Electronic Device-Related TR

As cardiac implantable electronic device (CIED) implantation rate has grown exponentially over the last few decades, the prevalence of CIED-related TR (a well-known potential complication following device implantation) currently ranges between 8–10% [19,20].

Despite being previously considered as a cause of primary TR, CIED-related TR and a worsening of pre-existing TR after CIED implantation are now recognized as separate clinical entities, as they have been associated with increased risk of heart failure and mortality in patients with CIED [21]. Progression of CIED-related TR results in right heart remodeling, with increased RA and RV volumes and subsequent decreased RV function [21].

Pathophysiological mechanisms determining CIED-related TR can be distinguished into implantation-related (which include sub-valvular entrapment of the lead, leaflet perforation, or impingement), pacing-related (as continuous pacing determines remodeling and mechanical biventricular dyssynchrony), and device-related (due to leaflet or sub-valvular apparatus fibrosis, scar formation, lead-induced endocarditis, and leaflet avulsion during lead extraction) [21,22]. The most common mechanism of CIED-related TR is leaflet impingement (39% of overall CIED-related TR), due to mechanical interference of the lead with the normal valve excursion and leaflet coaptation [23].

## 3. Treatment Options for TV Repair

### 3.1. Surgery

Surgical repair remains the recommended intervention in patients with low surgical risk in those undergoing open-heart surgery for revascularization or left-sided valve disease and in symptomatic patients with signs of right heart dysfunction or in patients with mild-to-moderate TR and annulus dilatation [24]. In particular, isolated TV surgery for severe TR in symptomatic patients with primary TR is a class I-C and class IIa-B recommendation in the European and the American guidelines, respectively [24,25]. Nonetheless, due to slow TR progression, patients tend to remain asymptomatic until a late stage, and, when evaluated for isolated TR surgery, they commonly present with high surgical risk due to impaired right heart function and several comorbidities, often including advanced age [26]. TV TEER might therefore be most beneficial in patients at moderate to high risk for surgery [27].

The TRI-SCORE has recently been proposed to predict mortality after isolated TV surgery in patients with TR and helps discriminate between patients who might benefit from TV surgery (TRI-SCORE < 8 points) and those at increased surgical risk (TRI-SCORE > 8 points) by taking into consideration clinical, biological, and echocardiographic factors (Table 2) (http://www.tri-score.com, accessed on 28 November 2024) [28].

### 3.2. Tricuspid TEER

The Triclip© device (Abbott, Santa Clara, CA) was developed from the pre-existing Mitraclip© system (Abbott, Santa Clara, CA) and redesigned and adapted for the TV. It includes a steerable guide catheter (SGC) to maneuver the system in the right heart and a clip delivery system [29]. Four different Triclip devices are currently available: NT, NTW, XT, and XTW, and the system allows for independent grasping of the TV leaflets [30].

The procedure is performed under general anesthesia and guided by transesophageal echocardiography (TEE) or fluoroscopy, and, in some centers, under conscious sedation, guided by intracardiac echocardiography (ICE).

The safety and efficacy of the Triclip device have been demonstrated in the international, randomized TRILUMINATE trial, which randomized 350 patients with severe TR to receive either medical therapy (control group) or percutaneous treatment (intervention group) [31]. The results for the primary endpoint (a hierarchical composite that included death from any cause or tricuspid valve surgery; hospitalization for heart failure; and an improvement in quality of life at 1-year follow-up) favored the TEER group (mainly driven by improvements in the Kansas City Cardiomyopathy Questionnaire [KCCQ] score) (a win ratio of 1.48; 95% CI 1.06–2.13; *p* = 0.02). Triclip was also confirmed to be safe, with 98.3% of patients free from major complications at 30 days [31]. A major limitation was the fact that the trial was open label, introducing potential interpretation bias: to reduce such bias, follow-up evaluations were blinded, except for the echocardiographic assessments [31].

In a recent systematic review and meta-analysis of 19 studies (observational or single-arm trials) including a total of 991 patients undergoing isolated transcatheter TV repair with several different devices, Alperi et al. reported a significant reduction of ≥severe TR (risk ratio [RR] 0.33; 95% CI 0.26–0.42; *p* < 0.001), vena contracta (VC) width (mean difference [MD] 5.9 mm; 95% CI 4–7.9; *p* < 0.001), RV end-diastolic diameter (MD 3.5 mm; 95% CI 2.5–4.5; *p* < 0.001), and New York Heart Association (NYHA) class III or IV at follow-up (RR 0.32; 95% CI 0.27–0.37; *p* < 0.001). Of note, bleeding complications and residual severe TR were numerically higher in the annuloplasty-like group compared with edge-to-edge repair (13.3% vs. 3.8% for bleeding and 40.4% vs. 27.9% for residual severe TR) [32].

## 4. Pre-Procedural Echo Imaging

Appropriate patient selection is crucial for the success of TV TEER. Pre-procedural transthoracic (TTE) and TEE assessment of TR aims to evaluate TR etiology, severity, location, and transvalvular diastolic peak and mean gradient (to exclude TV stenosis). Assessment of RA dimensions, RV function, and pulmonary hypertension are important in the pre-procedural evaluation, as they might impact patients’ morbidity and mortality [33].

Pre-procedurally, image quality of TEE should be evaluated both with the patient in the left lateral decubitus and supine positions, as the latter corresponds to the intraoperative position.

TV imaging can be challenging due to its anterior position in the chest cavity and far proximity from the TEE probe, possible acoustic shadowing from cardiac prosthetic material, device leads, interatrial septal hypertrophy, or mitral and/or aortic calcifications [34,35].

**Figure 2:**
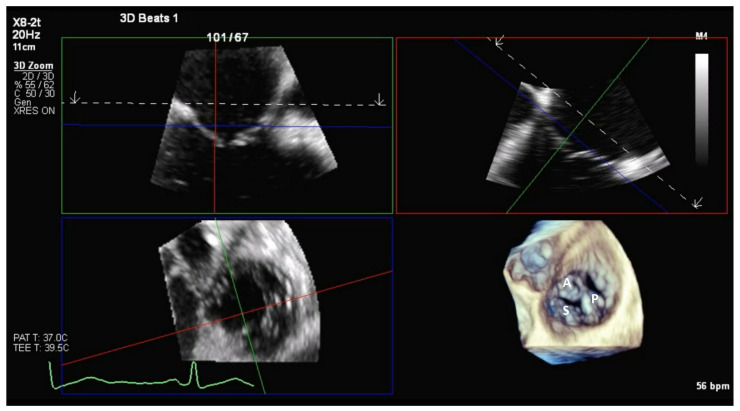
3D MPR showing a trileaflet tricuspid valve. To improve spatial orientation, landmark anatomical surrounding structures have been included in the dataset. MPR, multiplanar reconstruction; A, anterior leaflet; P, posterior leaflet; S, septal leaflet.

**Figure 3:**
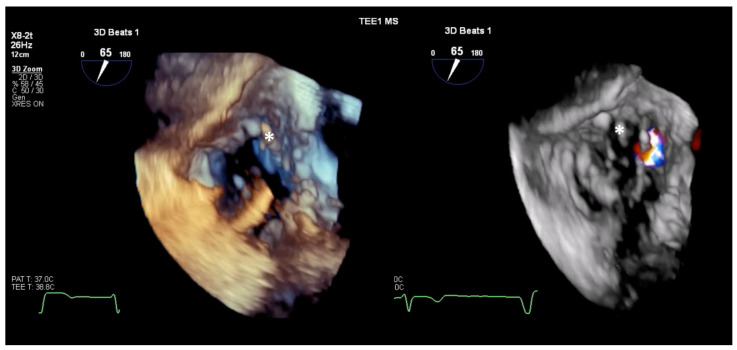
3D-zoom, en-face view of the TV, with and without color, showing a restricted septal leaflet due to CIED lead (white star), causing TR. CIED, cardiac implantable electronic device; TR, tricuspid regurgitation; TV, tricuspid valve.

These limitations can be partially overcome with the use of 3D-echo, which provides a more intuitive understanding of the complex anatomy of the TV and its spatial relationship with the surrounding structures (Figure 2) [36]. Multiplanar reconstruction from a 3D data set can be performed in real time or offline for better morphologic and anatomic assessment, especially to measure the TV area and leaflet lengths. Moreover, 3D imaging of the TV can help determine the location of CIED leads and any interference with the TV apparatus (Figure 3) [34].

### 4.1. 2D Echocardiography

2D color Doppler imaging has been fundamental in valve disease severity assessment since the dawn of 2D-echo. Nonetheless, while it is useful in identifying small jets of TR, it might underestimate eccentric jets, potentially leading to the suboptimal quantification of TR severity [37]. In the setting of more than minimal/mild regurgitation, a quantitative approach is advised using VC width, a proximal isovelocity surface area (PISA), and a derived effective regurgitant orifice area (EROA) [37]. VC width refers to the narrowest regurgitant flow diameter beyond the flow convergence region and before the expansion of the turbulent regurgitant jet [38]. The PISA method is the most recommended quantitative approach used to evaluate TR severity: it is based on the law of mass conservation and the hemodynamic principle that blood flow across a circular orifice forms concentric hemispheres. The goal of the PISA method is to calculate the EROA, as flow across the hemispheric surfaces equals the flow across the EROA [39]. TR is considered severe if the VC width is ≥7 mm, PISA EROA is ≥0.40 cm^2^, and the regurgitant volume is ≥45 mL [40].

The 2D quantitative approach has several limitations: for example, the dimension of the VC width often varies greatly depending on the view used to assess the regurgitant jet. Additionally, it is well known that the cross-sectional shape of the VC is often an ellipsoid; therefore, antero-posterior and septal-lateral VC width often yield different values [41]. Among others, the limitations of the PISA method include a lack of angle correction, variation of the hemispheric surfaces during the cardiac cycle, and the fact that in eccentric, wall-hugging jets, EROA calculated with the PISA method may underestimate the severity of TR [42,43]. Moreover, right heart pressures are usually lower than left heart pressures, except in the case of moderate/severe pulmonary hypertension, therefore affecting PISA method analysis and regurgitant flow assessment [44]. It should also be noted that incorrect evaluation of the TR degree may cause relevant mistakes in the calculation of systolic pulmonary artery pressure (sPAP): in the case of severe TR due to RV dilatation, for instance, the pronounced enlargement of the effective area of the regurgitating orifice may cause a reduction of TR velocity (TRV) and “truncation” of the continuous wave (CW)-Doppler spectrum of TR, with consequent underestimation of sPAP. Moreover, a small amount of TR could lead to an incomplete Doppler spectrum, with consequent underestimation of sPAP, especially in patients with chronic obstructive pulmonary disease (COPD) and/or advanced lung disease [45,46,47].

### 4.2. 3D Echocardiography

**Figure 4:**
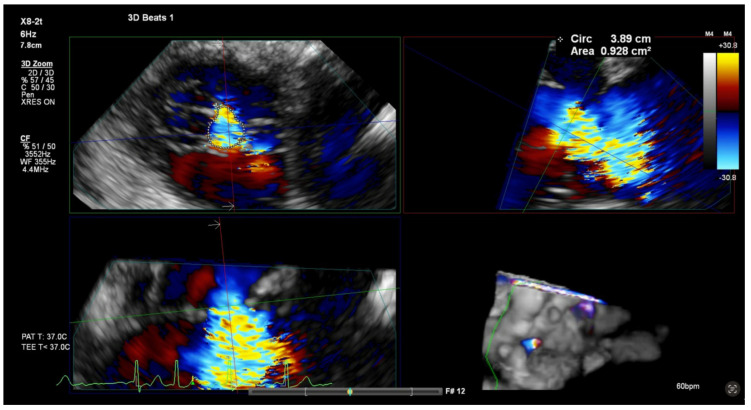
3D-TEE MPR to measure the VCA. After locking the orthogonal planes, the green plane is positioned at the level of the VC width in the red and blue plane. After careful adjustment, the VCA can be visualized and can be measured by direct planimetry in the green box. TEE, transesophageal echocardiography; MPR, multiplanar reconstruction; VC, vena contracta; VCA, vena contracta area.

Several of the limitations of 2D-echo can be overcome by using 3D imaging. As accurate and reproducible assessment of TR severity impacts clinical decision and management, a more precise interrogation of the TV using a 3D volumetric data set to process and directly measure the VC area (VCA) is recommended (Figure 4) [48].

To achieve the best spatial resolution, it is important that the TV is located in the center of the pyramidal volume acquisition. Acquisition on TTE or TEE can be completed from any acoustic window from an optimized 2D-echo image. Multibeat volume set acquisition can increase the frame rate and resolution, but often at the expense of stitch artifacts from arrhythmias or respiration. Breath holds, if the patient is intubated, can be performed to minimize these artifacts [49]. Frame rates greater than 12–15 Hz are recommended; the 3D sector width and height can be decreased to include only relevant structures. Additionally, it is essential that the TV 3D-echo acquisition includes some anatomical landmarks (such as the aorta) to help identify leaflets. The anterior TV leaflet abuts the RV outflow tract (RVOT), the septal is adjacent to the interventricular septum, and the posterior leaflet extends from the anterior papillary muscle to the postero-septal commissure, adjacent to the inferior wall of the RV [49].

3D-guided planimetry of VCA should be performed in the short-axis plane using multiplanar reconstruction (MPR) from a 3D volume set (Figure 4). “Locking” the planes allows correct orthogonal orientation while aligning with the VC in MPR and avoids over- or underestimating the area by measuring at non-perpendicular angles. 3D VCA assessment has shown incremental and independent diagnostic value for severe TR, since it does not rely on any geometric assumption of the EROA [50]: notably, a 3D VCA between 75–94 mm^2^ indicates severe TR, between 95-114 mm^2^ indicates massive TR, and ≥115 mm^2^ denotes torrential TR (Table 3) [44,51].

Furthermore, it is important to note that the tricuspid annulus is a non-planar structure with an elliptical saddle or ovoid shape and a variable size across the cardiac cycle [52,53]. When right heart dilatation occurs, the tricuspid annulus dilates along the RV free wall, and its shape becomes more planar and circular [54]. These geometrical changes are difficult to assess with 2D-echo; therefore, 3D assessment is imperative when evaluating such a highly dynamic structure.

Despite its many advantages, 3D-echo still has some limitations, including the suboptimal tissue characterization and evaluation of leaflet thickness, as 3D artifacts might make them appear thicker than they really are (blurring or amplification) [49].

## 5. Patient Selection for Percutaneous Repair

Patients who continue to be symptomatic despite maximal medical therapy should be assessed by the heart team for their eligibility for surgery or the TV TEER procedure. A number of parameters are evaluated to determine if a patient’s anatomy is suitable for a percutaneous intervention. Favorable anatomy is defined by trileaflet morphology, small leaflet coaptation gaps (<7 mm), an anteroseptal jet origin, adequate leaflet length (>10 mm), localized degenerative (prolapse or flail) pathology, and no CIED lead interaction [55]. Feasible anatomy includes non tri-leaflet morphology, a coaptation gap of up to 8.5 mm, a postero-septal jet location, and CIED-related TR [56].

Small coaptation gaps and anteroseptal jets are associated with better procedural outcomes [57]. However, patients with atrial TR usually have large coaptation gaps from annular dilatation; in these patients, TEER can still be feasible, but multiple clips are usually required. A “zipping technique” might be needed, with the first clip placed at the anteroseptal commissure, bringing together the leaflets and continuing clipping along the anterior-septal leaflet coaptation plane to reduce the gap [58].

As shown by Russo et al. in a cohort of 298 patients from the TriValve Registry, procedural success was similar between patients with atrial vs. ventricular TR (80% vs. 83%, respectively, *p* = 0.56), and TEER was effective in reducing TR in both groups. At 12-month follow-up, survival was significantly higher in the atrial vs. ventricular TR cohort (91% vs. 72%, log-rank *p* = 0.02), with ventricular TR being an independent predictor of mortality at multivariable analysis [59].

## 6. Intraprocedural Guidance

### 6.1. TEE-Guided Tricuspid TEER

Intraprocedural imaging begins with reassessment of the degree of TR and identification of the TEE views with the best image quality [60]. If imaging quality is suboptimal, a blood pressure cuff can be inflated under the patient’s right shoulder to slightly tilt the patient’s chest cavity towards the left while supine. Also, reducing the patient’s tidal volumes will reduce the cardiac translation from respiration.

**Figure 5:**
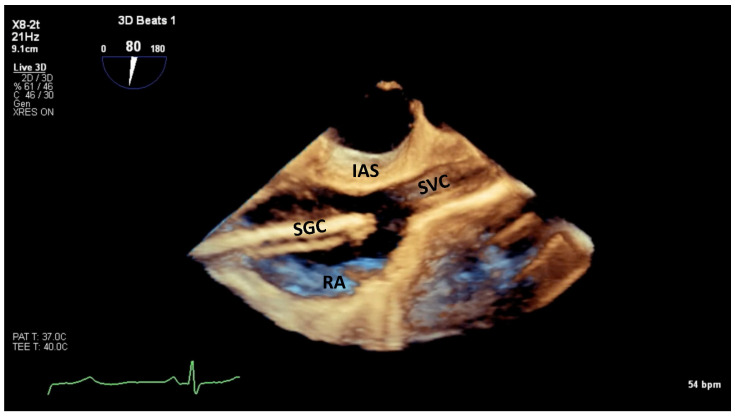
Live 3D helps maneuver the SGC inside the RA. IAS, interatrial septum; SGC, steerable guide catheter; RA, right atrium; SVC, superior vena cava.

**Figure 6:**
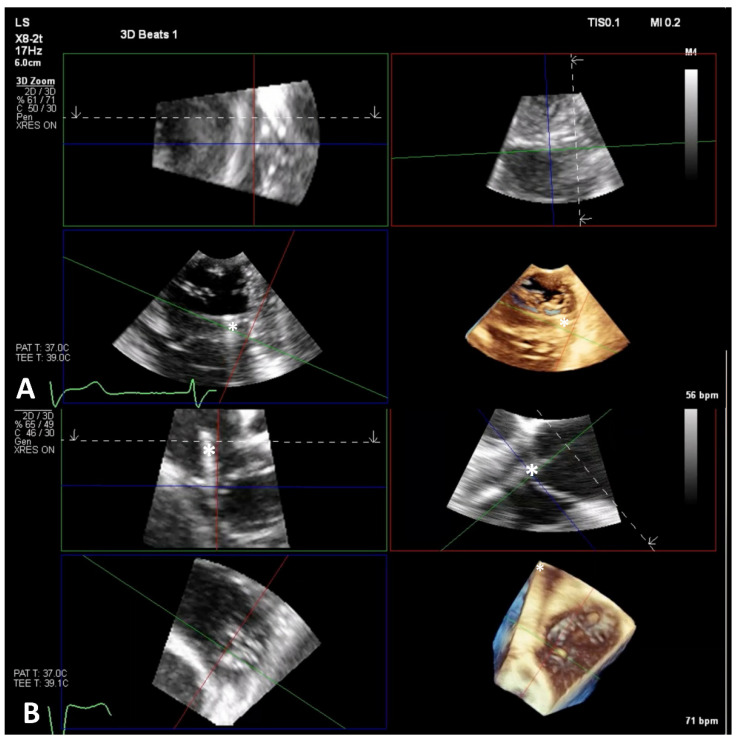
3D-zoom MPR to orient the Triclip (white star). Panel (**A**): The green and red planes indicate the designated location between the anterior and septal leaflets. Panel (**B**): Once the optimal site of clip deployment has been established, 3D MPR helps to advance the Triclip across the TV annulus plane. MPR, multiplanar reconstruction; TV, tricuspid valve.

After femoral venous access is obtained, the SGC is introduced into the right atrium over a guide wire. Through the guide, the Triclip delivery system is advanced away from the atrial septum and directed down towards the TV using TEE guidance, mainly in the bicaval view (90–110°). It is important to avoid entanglement in the Chiari Network or the Eustachian valve and avoid the right atrial appendage, maneuvering the clip towards the TV annular plane (Figure 5). The use of live 3D MPR allows visualization of the clip and valve leaflets in multiple orthogonal planes, eliminating the need to manipulate the probe and minimizing trauma to the patient’s esophagus [61]. 3D MPR planes are adjusted to keep the device in view relative to the TV, and gripper arms are checked to ensure adequate function. In cases of challenging anatomies or non-clear anatomical conformation of the TV apparatus, 3D MPR is of fundamental importance to assess valve leaflets, the sub-valvular apparatus, and the chordae tendinae. Orientation of the clip perpendicular to the leaflet coaptation plane is usually performed using an MPR data set from the best imaging window (Figure 6) or from the transgastric short axis of the TV [60].

**Figure 7:**
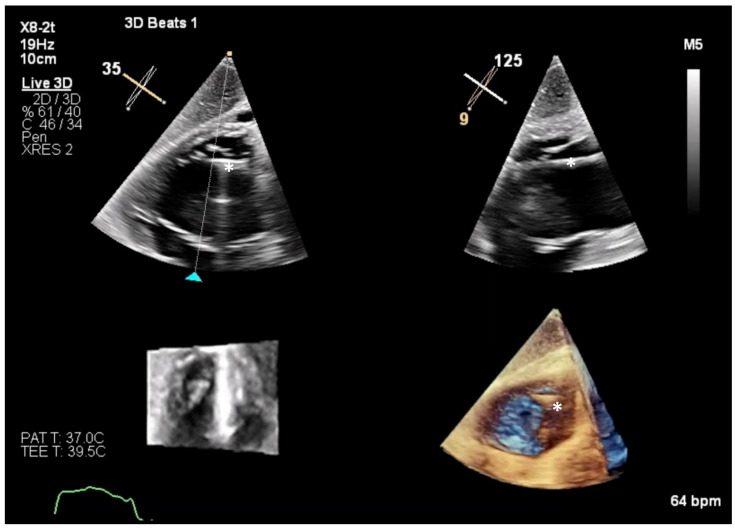
Live 3D helps to continuously monitor the advancement of the Triclip system (white star) across the TV annulus plane. Once the Triclip system has crossed the valve plane, it is crucial to reassess adequate orientation, as septal or lateral dive can occur while advancing the system in the RV. TV, tricuspid valve; RV, right ventricle.

Once the optimal site of clip deployment has been established, the delivery system is advanced across the TV, and clip orientation is followed by MPR imaging (Figure 7) and fluoroscopy to ensure no rotation has occurred and the clip trajectory remains perpendicular to the annulus, avoiding septal or lateral dives. It might be helpful to reduce the 3D gain to dropout TV leaflets and allow assessment of the clip positioning in the RV [60,61].

**Figure 8:**
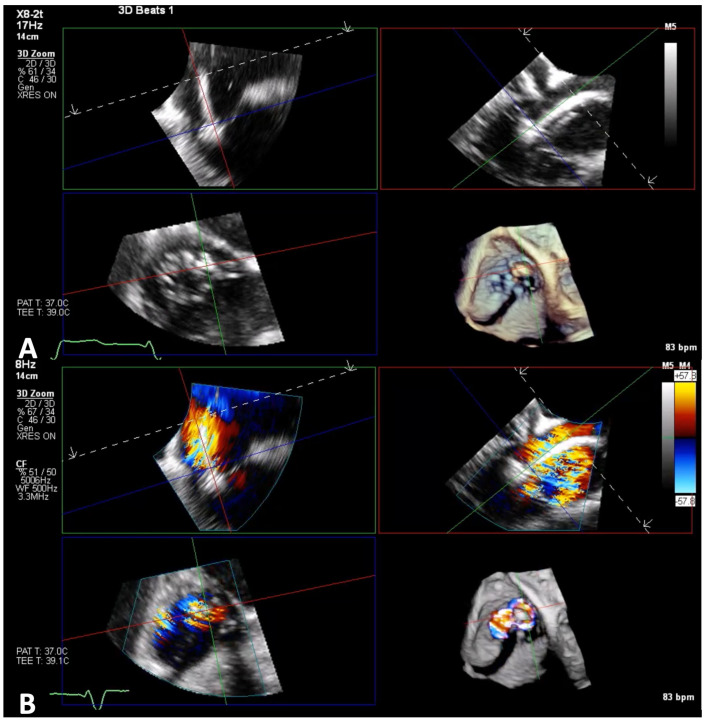
3D-zoom MPR to check adequate grasping and effective TR reduction. Panel (**A**): As shown in the orthogonal planes, both leaflets were grasped with good insertion. Panel (**B**): Despite a good grasping, TR was not sufficiently reduced. MPR, multiplanar reconstruction; TR, tricuspid regurgitation.

**Figure 9:**
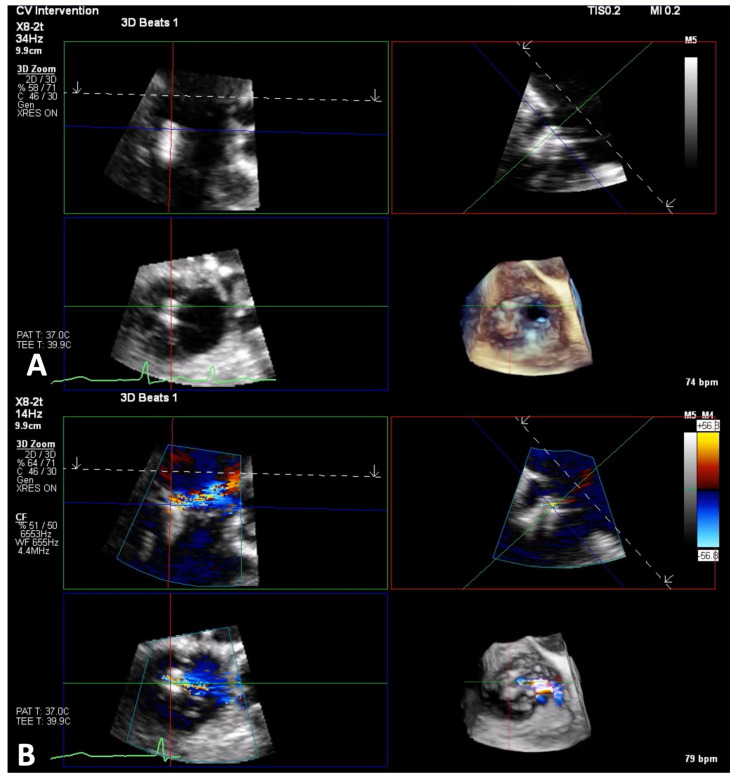
3D-zoom MPR to assess TV repair result. Panel (**A**): After deployment, Triclip looks stable and adequately attached to both leaflets. Panel (**B**): 3D color shows a reduction in TR, with only mild residual insufficiency. TV, tricuspid valve; TR: tricuspid regurgitation.

The opened clip is pulled back towards the tricuspid valve leaflets under TEE guidance towards the intended TV leaflet target. Once both leaflets are seen ”bouncing” on the clip arms, the grippers are dropped. If there is uncertainty regarding the appropriate leaflet insertion, independent grippers can be lifted to again visualize the amount of leaflet on the clip arm. When satisfactory leaflet insertion has been determined, the clip arms can be closed and color Doppler can be used to check the degree of regurgitation reduction (Figure 8). If both the proceduralist and the imager are happy with clip location, grasping, and consequent TR reduction, the clip is deployed (Figure 9). Inappropriate grasping may result in single-leaflet device attachment (a clip attached to only one leaflet) or clip embolization (a clip attached to no leaflets), potentially leading to catastrophic consequences [62]. Residual TR is reassessed after deployment, and if significant TR remains, the location of a second clip is determined.

3D-echo is fundamental in evaluating residual TR. Using biplane mode, the exact location of the residual leak can be identified in the inflow view (60–90°), and the leaflet length at that location can be assessed. Measurement of a direct VCA and residual TV area can be performed using MPR [63]. If subsequent clips are required, sweeping through the TV leaflets in 3D MPR will identify the exact location of the subsequent device [64].

Intracardiac echocardiography (ICE) imaging can be used when TEE imaging is suboptimal or to supplement TEE by obtaining a different perspective on the valve.

### 6.2. ICE-Guided TEER

**Figure 10:**
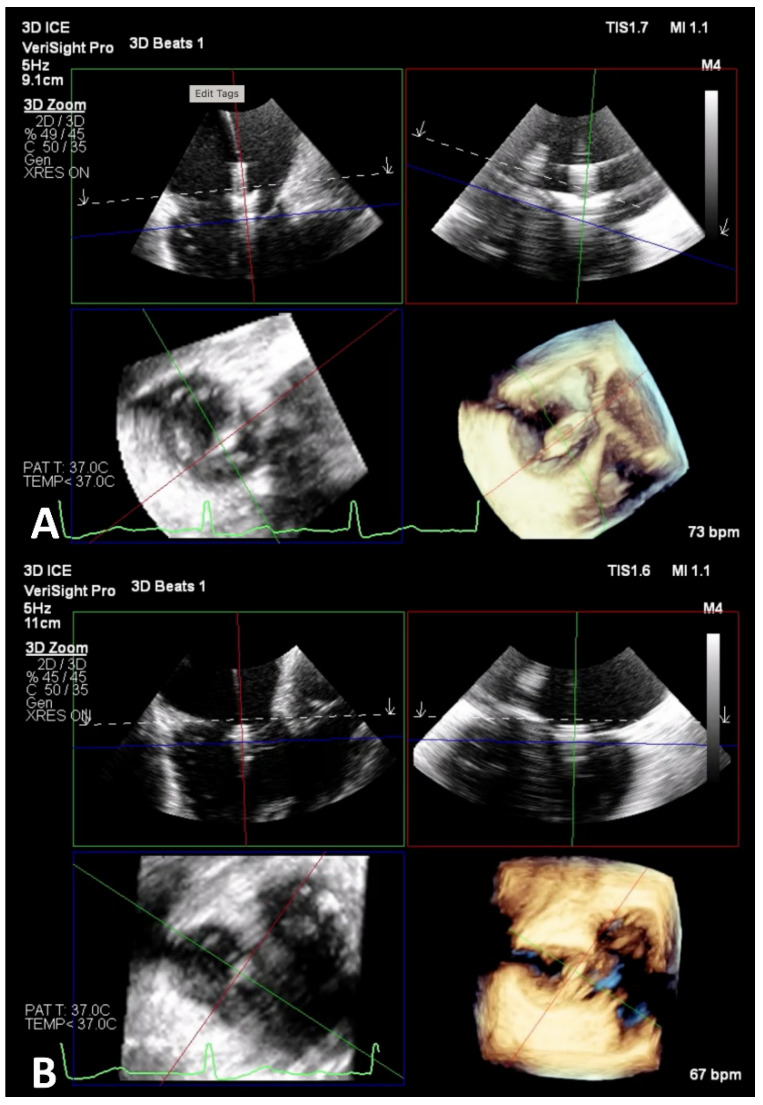
Adjunctive 3D ICE imaging during Triclip procedure. Panel (**A**): The proximity of the ICE catheter to the TV plane allows for a better visualization of the TV leaflets. Panel (**B**): 3D ICE is a supplementary imaging modality that is helpful during leaflet grasping and determining appropriate leaflet insertion. ICE, intracardiac echocardiography; TEE, transesophageal echocardiogram; TV, tricuspid valve.

ICE positioning in the RA allows for imaging guidance during TEER procedures. The ICE catheter is positioned in the RA but not directly behind the SGC, where the inflow-outflow view of the TV can be seen best. 3D ICE allows for biplane imaging along with building 3D volume sets. Manipulation of the ICE catheter is performed by the proceduralist with console knobology, usually performed by the imager, sonographer, or company representative. Although 3D TEE remains the gold standard for intra-procedural guidance for TV TEER, the use of combined TEE and ICE imaging has its advantages. ICE gives another perspective when grasping and checking for leaflet insertion into the clip (Figure 10), which is especially helpful in patients with limited TEE images [65]. Ideal grasping encompasses at least 7 mm of leaflet issue inserted into the clip arms, with the leaflets lying flat into the device [66].

The limitations of 3D ICE include its cost and single-use nature [67]. Moreover, adequate training is needed by both the proceduralist and the imager in order to obtain the necessary views to optimize the catheter’s maneuvering. To this aim, communication is critical to coordinate the manual steering of the catheter and digital processing of the images while decreasing procedural time [68].

The intraprocedural steps for ICE-guided TEER have been extensively described by Hamid et al. [68].

## 7. Future Directions

Manipulation of the TEE probe at multiple levels of the esophagus during procedures such as TV TEER can lead to direct trauma or thermal injury to the mucosa of the esophagus or stomach [69]. 3D MPR imaging reduces probe manipulation, reducing the risk of trauma [70]. Moreover, a mini TEE probe has been proven safe and feasible in guiding structural heart interventions under conscious sedation with no need for general anesthesia [71].

3D ICE-only imaging could represent a safe alternative to imaging guidance, avoiding the esophagus altogether once the resolution of the technology improves. This would allow for procedures to be performed under conscious sedation or local anesthesia, bypassing general anesthesia in frail TR patients presenting with severe comorbidities [67]. Nevertheless, further advancements in ICE technology are still needed in order for TV TEER to be routinely ICE solo-guided [66].

In patients with complex anatomies, real-time echo-fluoroscopy fusion imaging with integrated echo overlay on fluoroscopy can support the proceduralist in navigating the Triclip device in the right heart and improve spatial orientation and temporal resolution [72]. Customized anatomic landmarks can be set, aiming to effectively maneuver the SGC within the RA and towards the TV plane [73]. Advantages of fusion imaging include reduction in radiation exposure, a contrast dose, and a decrement in procedural time, compared to conventional imaging techniques [73].

The future of TV TEER imaging looks very promising and full of exciting advancements; nonetheless, further studies are needed to confirm the added clinical value of such emerging imaging tools.

## Figures and Tables

**Table 1:** Current classification scheme of tricuspid valve nomenclature, based on the number of leaflets and their incidence. Adapted with permission from Hahn et al. [2].

Number of Leaflets	3 Leaflets	2 Leaflets	4 Leaflets			>4 Leaflets
**Type**	Type I	Type II	Type IIIA (2 anterior leaflets)	Type IIIB (2 posterior leaflets)	Type IIIC (2 septal leaflets)	Type IV
**Incidence**	54%	5%	3%	32%	4%	2%

**Table 2:** Clinical, biological, and echocardiographic parameters included in the TRI-SCORE calculation. A total score > 8 points denotes an increased surgical risk for the patient. A total score < 8 indicates that the patient will benefit from TV surgery (available at http://www.tri-score.com) [28].

Risk Factors Included in the TRI-SCORE Calculation	
Age ≥ 70 years	1 point
NYHA Class III or IV	1 point
LVEF < 60%	1 point
At least moderate RV dysfunction	1 point
Right-side heart failure	2 points
Daily furosemide ≥ 125 mg	2 points
Filtration rate < 30 mL/min	2 points
Elevated total bilirubin	2 points

LVEF, left ventricular ejection fraction; NYHA, New York Heart Association; RV, right ventricle; TV, tricuspid valve.

**Table 3:** Summary of the main 2D and 3D echocardiographic parameters to assess TR severity. Adapted with permission from Hahn et al. and Go et al. [44,51].

	Mild TR	Moderate TR	Severe TR	Massive TR	Torrential TR
**VC width**	<3 mm	3–6.9 mm	7–13 mm	14–20 mm	≥21 mm
**EROA calculated with PISA method**	<20 mm^2^	20–39 mm^2^	40–59 mm^2^	60–79 mm^2^	≥80 mm^2^
**Regurgitant Volume calculated with PISA method**	<30 mL	30–44 mL	45–59 mL	60–74 mL	≥75 mL
**3D VCA or Quantitative Doppler EROA**	-	-	75–94 mm^2^	95–114 mm^2^	≥115 mm^2^

EROA, effective regurgitant orifice area; PISA, proximal isovelocity surface area; TR, tricuspid regurgitation; VC, vena contracta; VCA, vena contracta area.

## Data Availability

No new data were created or analyzed in this study. Data sharing is not applicable to this article.
